# Genome and transcriptome profiling of spontaneous preterm birth phenotypes

**DOI:** 10.1038/s41598-022-04881-0

**Published:** 2022-01-19

**Authors:** Juhi K. Gupta, Angharad Care, Laura Goodfellow, Zarko Alfirevic, Bertram Müller-Myhsok, Ana Alfirevic

**Affiliations:** 1 Wolfson Centre for Personalised Medicine, Department of Pharmacology and Therapeutics, Institute of Systems, Molecular and Integrative Biology, University of Liverpool, Block A: Waterhouse Buildings, 1-5 Brownlow Street, Liverpool, L69 3GL UK; 2 Harris-Wellbeing Research Centre, University Department, Liverpool Women’s Hospital, Liverpool, L8 7SS UK; 3 Max Planck Institute of Psychiatry, 80804 Munich, Germany

**Keywords:** Genetics research, Genetics, Systems biology, Biomarkers

## Abstract

Preterm birth (PTB) occurs before 37 weeks of gestation. Risk factors include genetics and infection/inflammation. Different mechanisms have been reported for spontaneous preterm birth (SPTB) and preterm birth following preterm premature rupture of membranes (PPROM). This study aimed to identify early pregnancy biomarkers of SPTB and PPROM from the maternal genome and transcriptome. Pregnant women were recruited at the Liverpool Women’s Hospital. Pregnancy outcomes were categorised as SPTB, PPROM (≤ 34 weeks gestation, n = 53), high-risk term (HTERM, ≥ 37 weeks, n = 126) or low-risk (no history of SPTB/PPROM) term (LTERM, ≥ 39 weeks, n = 188). Blood samples were collected at 16 and 20 weeks gestation from which, genome (UK Biobank Axiom array) and transcriptome (Clariom D Human assay) data were acquired. PLINK and R were used to perform genetic association and differential expression analyses and expression quantitative trait loci (eQTL) mapping. Several significant molecular signatures were identified across the analyses in preterm cases. Genome-wide significant SNP rs14675645 (*ASTN1*) was associated with SPTB whereas microRNA-142 transcript and PPARG1-FOXP3 gene set were associated with PPROM at week 20 of gestation and is related to inflammation and immune response. This study has determined genomic and transcriptomic candidate biomarkers of SPTB and PPROM that require validation in diverse populations.

## Introduction

Preterm birth (PTB) occurs when an infant is born prior to 37 completed weeks of gestation and is a major public health issue. PTB is a multifactorial condition and is associated with a number of poor health outcomes in infants including cerebral palsy, problems with vision and hearing, poor motor skills, asthma, autism and increased metabolic and cardiovascular risks to health^1,2^. Spontaneous PTB (sPTB) accounts for approximately two-thirds of all PTB and describes women that labour early, often for no clear reason. The other one-third are healthcare-provider initiated, usually in response to severe maternal or fetal disease (e.g. pre-eclampsia or severe intrauterine growth restriction) and will not be considered further in this study. A higher rate of infant morbidity is associated with ≤ 34 weeks of gestation and therefore this was applied as the threshold for sPTB in this study^3^.

Studies have shown that a familial hereditary element increases the risk of early labour. Women who were born preterm were more likely have a preterm delivery, and a women who had an obstetric history of sPTB is more likely to have a subsequent sPTB^4–7^. Both environmental factors (such as infection or lifestyle choices) and genetic factors are risks also associated with sPTB^8,9^. The role of infection pathway and associated genes, interleukins or tumour necrosis factors (TNF) has been reported in many studies, suggesting that the maternal genome should be screened for potential genetic biomarkers^10–14^. A large PTB GWAS study by Zhang et al.^15^, consisting of over 43,000 women, identified *EBF1* variant (Early B-cell factor 1, a transcription factor) associated with an increased risk of PTB further supporting a genetic cause involving immune response/inflammatory pathways.

Two clinical phenotypes of sPTB are (1) spontaneous preterm birth (SPTB) following the spontaneous onset of labour and (2) preterm premature rupture of membranes (PPROM) where the amniotic membrane spontaneously breaks and increases the risk of infection or preterm labour at a later date^16^. Despite obvious clinical differences, these two subgroups are often not differentiated in research studies when determining biomarkers for risk prediction, despite a universal acceptance that SPTB has multiple causes^17^. However, Capece et al.^18^ conducted pathway analysis of PTB genetic studies and proposed the role of immune and hormonal regulation in SPTB, versus the role of hematologic disorder, collagen metabolism, matrix degradation and local inflammation in PPROM. Differences in response to PTB preventative treatments, of women with a history of either PPROM or SPTB, in a subsequent pregnancy was described by Care et al.^19^, suggesting these women are different, and may have unique genetics influencing their response to known clinical treatments.

Recent PTB studies have identified the association of PTB with maternal microRNAs^20–24^. Short, non-coding microRNAs are key post-transcriptional regulators, regulating gene expression by destabilising mRNA and thereby repressing protein production^25,26^. Exploring expression quantitative trait loci (eQTL) can determine associations between SNPs and transcripts to gain functional insights on candidate biomarkers using linear models^27^.

No study has attempted to link genetic inheritance with gene expression in women with SPTB and PPROM. We have acquired and analysed genome-wide SNP data and transcriptome gene expression data from the same cohort of women collected simultaneously from maternal blood during pregnancy to perform eQTL mapping to determine potential biomarkers of spontaneous preterm birth.

To our knowledge, this is the first investigation to explore both genome-wide and transcriptome-wide profiles in prospectively collected, mid-trimester maternal blood samples (from a well-defined cohort, using a PTB cut-off of ≤ 34 weeks gestation), to determine biomarkers of spontaneous preterm birth phenotypes PPROM and SPTB.

## Results

**Figure 1:**
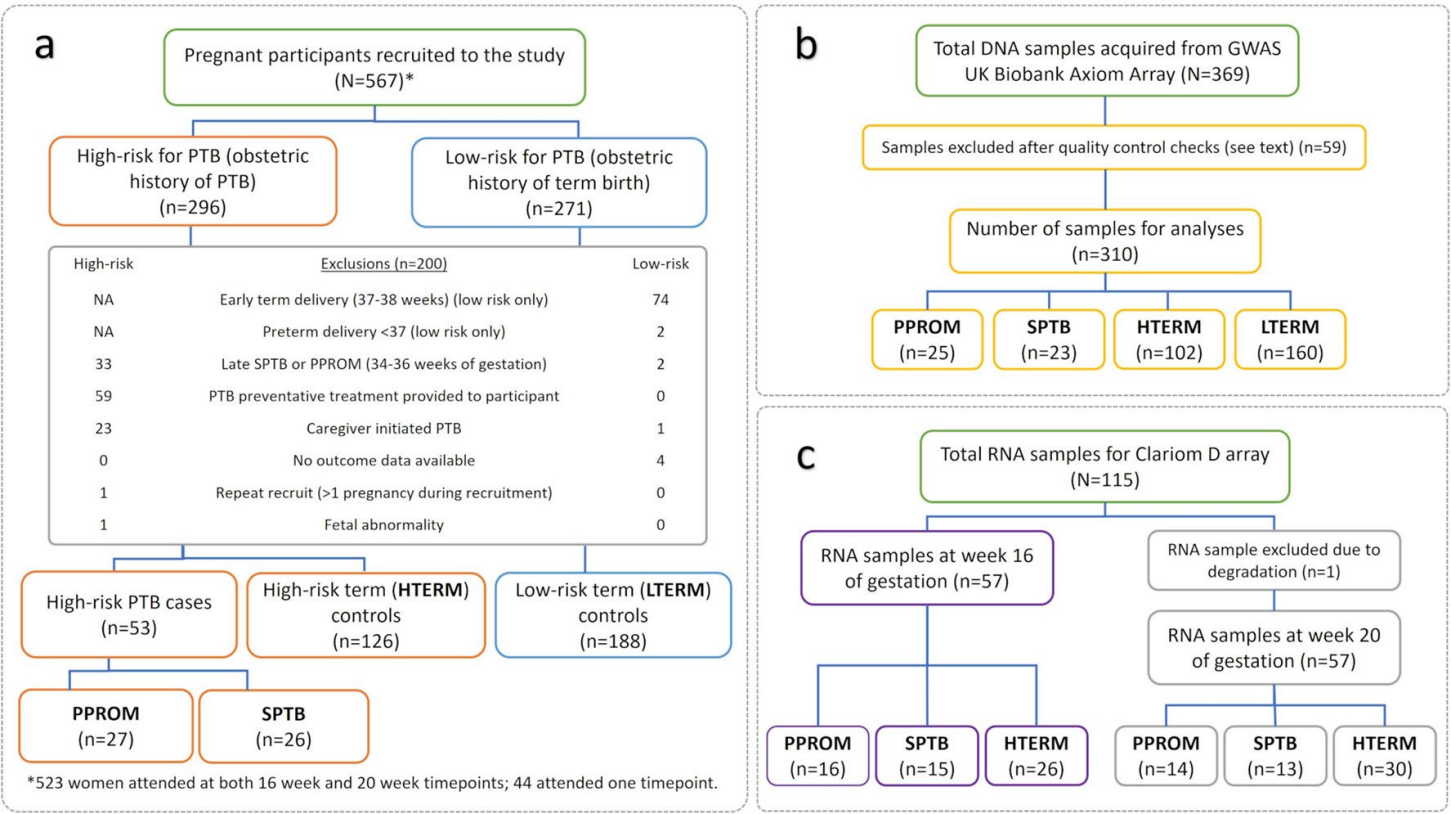
Singleton pregnant participants recruited to the Liverpool preterm birth study cohort. (**a**) Final number of women included in the analyses. (**b**) DNA was extracted from whole blood collected from the participants and genomic profiling (GWAS) was conducted on the Biobank Axiom™ array. (**c**) RNA extracted from whole blood collected from participants at 16 and/or 20 weeks of gestation was processed on the Clariom D array. *GWAS* genome-wide association study, *HTERM* high-risk term births, *LTERM* low-risk term births, *PPROM* preterm premature rupture of membranes, *PTB* preterm birth, *SPTB* spontaneous preterm birth.

Figure 1a summarises the number of women recruited to the study. A total of 310 DNA samples were included in GWAS analyses (Fig. 1b) and 114 RNA samples in gene expression analysis (Fig. 1c).

### GWAS SNP associations

GWAS analyses were conducted on the PTB phenotypes (cases), (1) SPTB and (2) PPROM against the term birth groups, (3) LTERM (recurrent term births only) and (4) HTERM (previous PTB with subsequent term birth).

### PPROM versus LTERM

**Figure 2:**
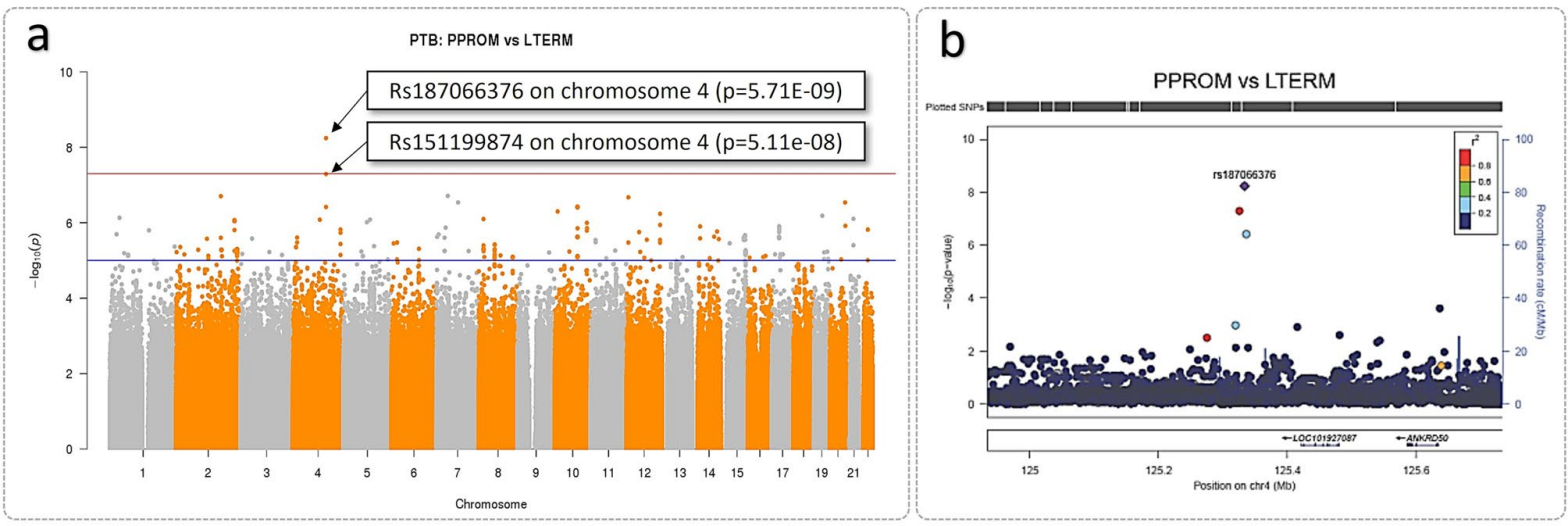
Genome-wide significant SNP identified from PPROM cases and LTERM controls GWAS analysis. (**a**) Manhattan plot of PPROM (n = 25) ≤ 34 weeks gestation and LTERM (n = 160) ≥ 39 weeks gestation GWAS analysis using Frequentist association test. Multi-dimensional scaling components 1 to 6 of the cohort were included as covariates. The upper red horizontal line displays the genome-wide significance threshold (p < 5 × 10^–8^) and the lower blue horizontal line represents an arbitrary suggestive threshold (p < 1 × 10^–5^). One SNP (rs187066376) exceeded genome-wide significance in this analysis on chromosome 4 (p = 5.71E−09). Figure generated using R package ‘qqman’^28^. (**b**) Regional plot of rs187066376 signal. Two SNPs are shown in linkage disequilibrium in red and blue. Figure produced using LocusZoom^29^.

PPROM versus LTERM analysis resulted in two genome-wide significant SNPs at chromosome 4, rs187066376 (p = 5.71e−09) and rs151199874 (p = 5.11e−08) both in non-coding region, LOC105377408 (Fig. 2a). SNP towers at chromosome 10 (rs34638554) and 12 (rs77423197) were further identified in non-coding regions (Fig. 2b).

### SPTB versus HTERM

**Figure 3:**
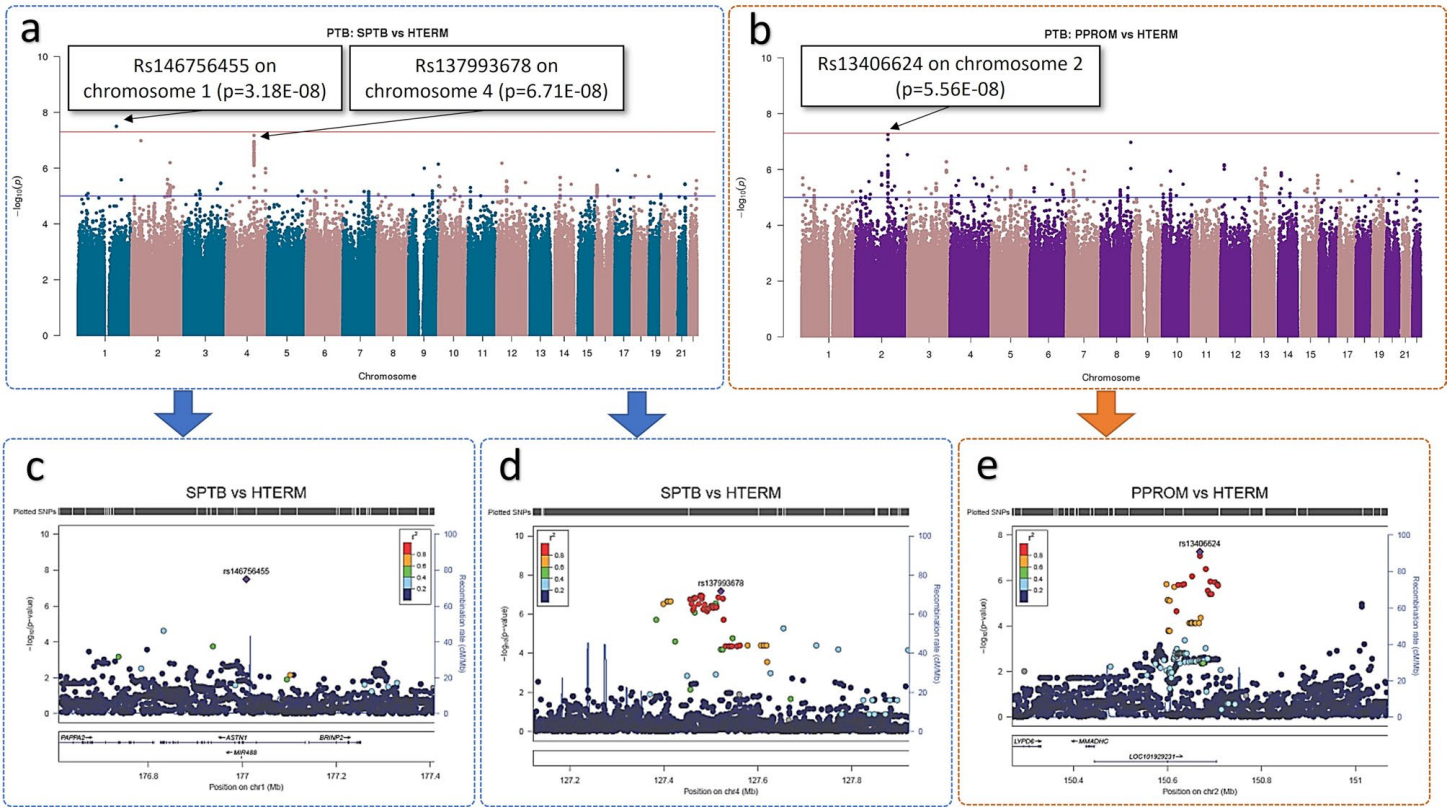
Manhattan plots of preterm versus term births GWAS analyses using Frequentist association test. (**a**) SPTB (n = 23) ≤ 34 weeks gestation and HTERM (n = 102) ≥ 37 weeks gestation and (**b**) PPROM (n = 25) ≤ 34 weeks gestation and HTERM (n = 102) ≥ 37 weeks gestation. Multi-dimensional scaling components 1 to 6 of the cohort were included as covariates. The upper red horizontal line displays the genome-wide significance threshold (p < 5 × 10^–8^) and the lower blue horizontal line represents an arbitrary suggestive threshold (p < 1 × 10^–5^). Manhattan plots were generated using R package ‘qqman’^28^. Regional plots: (**c**) genome-wide significant SNP (rs146756455) on chromosome 1 was identified as an intron variant in the gene, *ASTN1* (p = 3.18E−08) (SPTB versus HTERM); (**d**) rs137993678 approaching genome-wide significance on chromosome 4 (p = 6.71E−08) (SPTB versus HTERM). Several SNPs are in linkage disequilibrium; (**e**) rs13406624 on chromosome 2 was identified as a non-coding region, LINC01931 (or MMADHC-DT) (p = 5.56E−08) (PPROM versus HTERM). Regional plots were produced using LocusZoom^29^.

SPTB versus HTERM association analysis yielded a genome-wide significant SNP on chromosome 1 (rs146756455, p = 3.18E−08) and a suggestive SNP signal, close to genome-wide significance threshold, on chromosome 4 (Fig. 3a). The genome-wide significant SNP, rs146756455, is an intronic variant of *ASTN1* (p = 3.18E−08), which encodes astrotactin 1 (Fig. 3c). The association signal approaching genome-wide significance threshold at chromosome 4 (rs137993678) was not in a gene coding region (Fig. 3d).

### PPROM versus HTERM

No genome-significant SNPs resulted from the PPROM versus HTERM GWAS analysis. The SNP signal rs13406624 at chromosome 2 (p = 5.56E−08), approaching genome-wide significance, was in a non-coding region (Fig. 3b). Multiple SNPs were in the linkage disequilibrium (LD) with rs13406624 at chromosome 2 in non-coding RNA, LINC01931 (or MMADHC divergent transcript) (Fig. 3e).

### SPTB versus LTERM

Association analysis between SPTB and LTERM births yielded no genome-wide significant SNPs, however multiple signals were obtained (Supplementary Fig. S1, see Supplementary File 1). An association signal at chromosome 10 (rs9424165) obtained the lowest p-value is an intronic variant in *CAMK1D* (calcium/calmodulin-dependent protein kinase ID). Chromosome 1 rs2092868 is an intron variant in *NFIA* (Nuclear Factor I A) region, chromosome 2 signal (rs78202288) is a *MYO3B* (myosin IIIB) intron variant, chromosome 3 (rs61796814) signal is not in a coding region and chromosome 17 signal (rs150140114) is an intron variant in *AKAP10* (A-kinase anchor protein 10) region.

### LTERM versus sPTB cases

**Figure 4:**
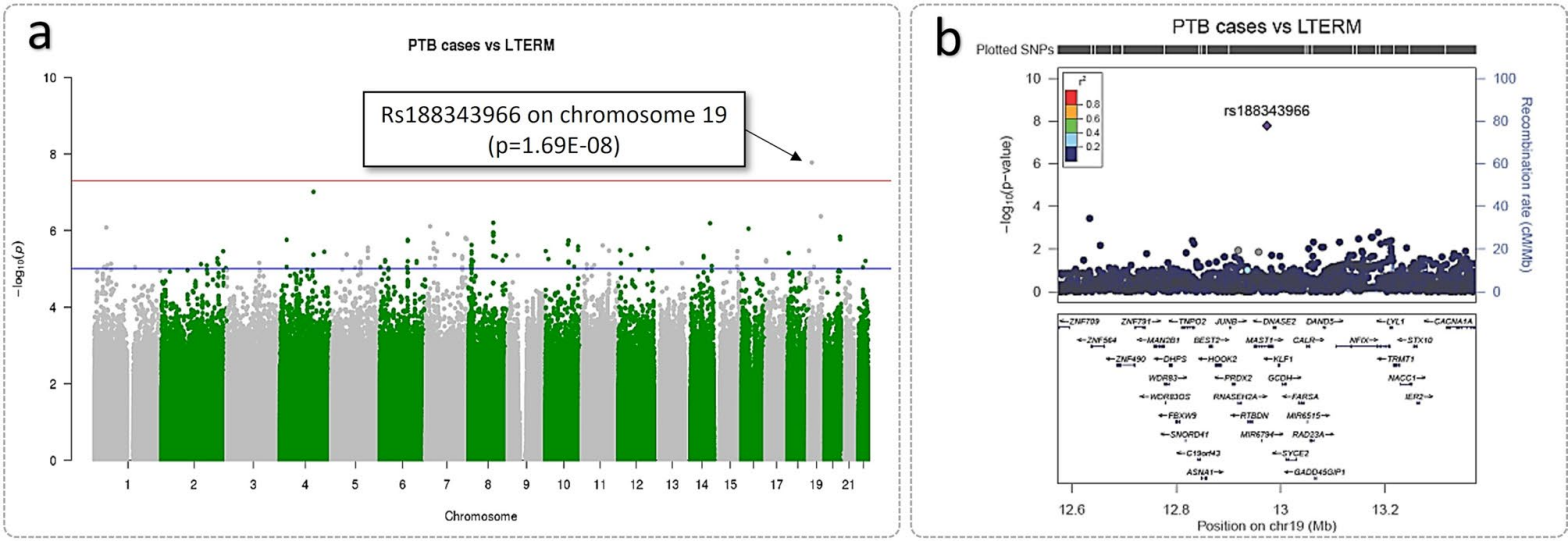
Genome-wide significant SNP identified from PTB cases and LTERM controls GWAS analysis. (**a**) Manhattan plot of all PTB cases (n = 48) ≤ 34 weeks gestation and LTERM (n = 160) ≥ 39 weeks gestation GWAS analysis using Frequentist association test. Multi-dimensional scaling components 1 to 6 of the cohort were included as covariates. The upper red horizontal line displays the genome-wide significance threshold (p < 5 × 10^–8^) and the lower blue horizontal line represents an arbitrary suggestive threshold (p < 1 × 10^–5^). One SNP (rs188343966) exceeded genome-wide significance in this analysis on chromosome 19 (p = 1.69E−08). Figure generated using R package ‘qqman’^28^. (**b**) Regional plot of rs188343966 signal. Figure produced using LocusZoom^29^.

Analysis of sPTB cases and LTERM highlighted a genome-wide significant SNP, rs188343966 (p = 1.69E−08, chr19:12973701), which is an intron variant in gene *MAST1* (Microtubule Associated Serine/Threonine Kinase 1) (Fig. 4a,b). No SNPs were identified in linkage disequilibrium with *MAST1* (rs188343966) (Fig. 4b).

### HTERM versus sPTB cases

No genome-wide significant SNPs were identified from the sPTB cases versus HTERM GWAS analysis (Supplementary Fig. S2). The lowest p-value signal on chromosome 16 (rs59159780, p = 4.14E−07) is not within a gene region (Supplementary Fig. S2).

### Differential gene expression analysis at week 16 of gestation

A total of 30,120 differentially expressed transcripts were identified at week 16 of gestation (p < 0.05), when analysing gene expression across SPTB versus HTERM, PPROM versus HTERM and SPTB versus PPROM. However none were significant at FDR p < 0.05 and did not reach log fold change threshold of < 1.5 or > 1.5.

### Differential gene expression analysis at week 20 of gestation

**Figure 5:**
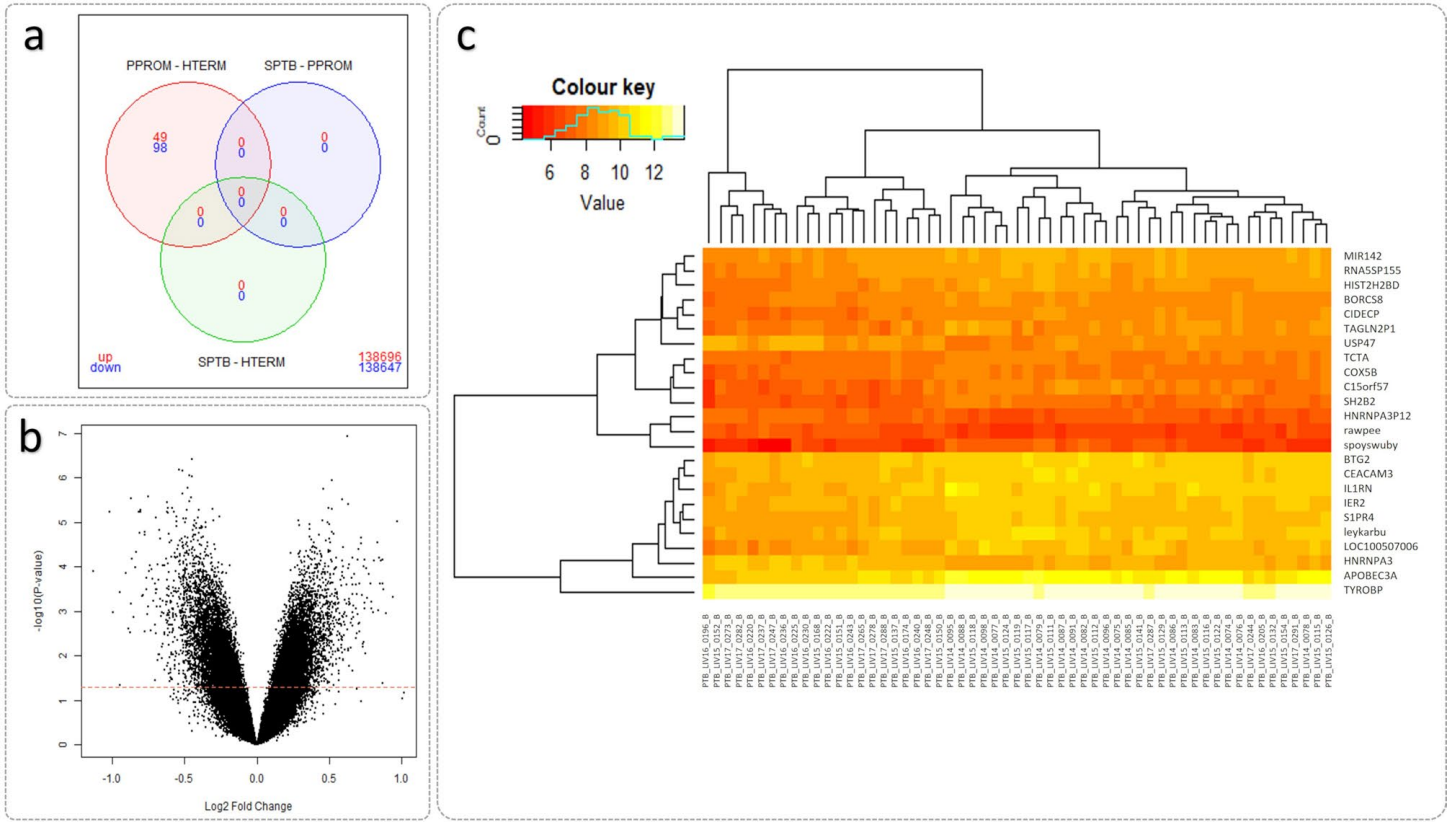
Significant DEGs identified from PPROM versus HTERM analysis at week 20 gestation of 57 maternal samples. (**a**) Venn diagram of 147 DEGs (49 upregulated and 98 downregulated) were significant at FDR p < 0.05. (**b**) Volcano plot of DEGs based on nominal p < 0.05. (**c**) Heatmap outlining 24 DEGs (with annotations available) that reached FDR p < 0.03. These figures were produced suing ‘limma’ R package^30^.

At week 20 of gestation, 147 differentially expressed genes (DEGs) (49 upregulated and 98 downregulated) were significant at FDR p < 0.05 for the PPROM versus HTERM analysis (Fig. 5a). Of these significant DEGs, none reached > 1.5 or < − 1.5 log fold change (Fig. 5b; Supplementary File 2) (Fig. 5c). An unknown transcript (Probe ID: TC0300009931.hg.1), with no annotation available, yielded the lowest p-value (p = 1.19E−07, FDR p = 0.02). Of the transcripts with annotations available, the top significant findings (FDR p < 0.03) are highlighted in Fig. 5c heatmap and summarised in Table 1. Full results from this analysis can be found in Supplementary File 2. No significant results were determined for SPTB versus HTERM or SPTB versus PPROM.

**Table 1:** Top 21 differentially expressed genes detected from PPROM versus HTERM expression analysis at week 20 of gestation.

ID	Symbol	Genename	logFC*	p	FDR P^†^
NR_029683	MIR142	MicroRNA 142	− 0.513	6.79E−07	0.022
NM_001862	COX5B	Cytochrome c oxidase subunit Vb	− 0.451	9.51E−07	0.022
ENST00000437410	HNRNPA3P12	Heterogeneous nuclear ribonucleoprotein A3 pseudogene 12	0.518	1.16E−06	0.023
NM_194247	HNRNPA3	Heterogeneous nuclear ribonucleoprotein A3	0.458	1.79E−06	0.028
NM_003775	S1PR4	Sphingosine-1-phosphate receptor 4	− 0.513	2.28E−06	0.028
NM_004907	IER2	Immediate early response 2	− 0.453	2.73E−06	0.028
NM_001080791	C15orf57	Chromosome 15 open reading frame 57	− 0.867	2.87E−06	0.028
NM_001173514	TYROBP	TYRO protein tyrosine kinase binding protein	− 0.466	3.40E−06	0.028
ENST00000411154	RNA5SP155	RNA, 5S ribosomal pseudogene 155	− 0.622	3.56E−06	0.028
NM_001282659	USP47	Ubiquitin specific peptidase 47	0.634	4.02E−06	0.028
NR_002786	CIDECP	Cell death-inducing DFFA-like effector c pseudogene	− 0.583	4.78E−06	0.028
NM_020979	SH2B2	SH2B adaptor protein 2	− 0.613	4.85E−06	0.028
NM_022171	TCTA	T-cell leukaemia translocation altered	− 0.305	4.93E−06	0.028
NM_006763	BTG2	BTG family, member 2	− 0.482	5.22E−06	0.028
NM_001145783	BORCS8	BLOC-1 related complex subunit 8	− 0.400	5.31E−06	0.028
OTTHUMT00000321237	APOBEC3A	Apolipoprotein B mRNA editing enzyme, catalytic polypeptide-like 3A	− 0.807	5.33E−06	0.028
OTTHUMT00000380415	TAGLN2P1	Transgelin 2 pseudogene 1	− 0.623	5.90E−06	0.028
NM_001277163	CEACAM3	Carcinoembryonic antigen-related cell adhesion molecule 3	− 0.575	5.93E−06	0.028
NM_000577	IL1RN	Interleukin 1 receptor antagonist	− 0.816	5.99E−06	0.028
NR_120420	LOC100507006	Uncharacterized LOC100507006	− 0.804	5.99E−06	0.028
OTTHUMT00000087130	HIST2H2BD	Histone cluster 2, H2bd (pseudogene)	− 0.617	6.28E−06	0.028

*logFC = log2 (fold change).

^†^Significant at FDR p < 0.03, not including 3 unspliced transcripts.

### Lysophosphatidic acid receptor pathway identified

Of the 147 significant DEGs detected for week 20 PPROM versus HTERM comparison, 119 were associated with gene symbols and uploaded on Reactome for pathway analysis. Lysosphingolipid and Lysophosphatidic acid (LPA) receptors pathway was determined as significant at p < 0.05 (p = 0.0096), however this was not significant at FDR p < 0.05.

### Gene set enrichment analysis at week 16 of gestation

Several significant gene sets from FUMA (FDR p < 0.05) were obtained: SPTB versus HTERM (n = 1864), SPTB versus PPROM (n = 372) and PPROM versus HTERM (n = 3148). Many of these were reported as ‘chemical and genetic perturbations’.

### Gene set enrichment analysis at week 20 of gestation

Similar significant gene sets as week 16 of gestation were also identified at week 20 (FDR p < 0.05): SPTB versus HTERM (n = 3705), SPTB versus PPROM (n = 70) and PPROM versus HTERM (n = 8469). However, the lowest p-value was determined for the gene set GSE37533 PPARG1-FOXP3-VS-FOXP3-TRANSDUCED-CD4-TCELL-DN (an ‘immunologic signature’) from the SPTB versus HTERM analysis, whereby 57 input genes overlapped (FDR p = 1.57E−13) (see Supplementary File 3). Peroxisome proliferator-activated receptor, or PPAR-gamma, are nuclear receptors involved with regulatory T cells, and FOXP3 (forkhead box P3) is part of a transcriptional factor family.

Comparison of SPTB versus HTERM DEGs to Genotype-Tissue Expression (GTEx) database determined a gene set (including 332 genes) that was significantly upregulated in whole blood at week 20 of gestation (FDR p = 0.021) (Supplementary Fig. S3, see Supplementary File 4). This included: *TNFRSF4, TNFRSF1B, IL1B, IL1RN, SLC11A1, HLA-V*, *HLA-G* and *MIR142*. Gene sets were also enriched in cervix/endocervix, ovary and vagina (but not significant at FDR p < 0.05).

### EQTL mapping at week 16 of gestation

From the GWAS analyses of each phenotype, a total of 876 SNPs reached suggestive threshold (p < 1 × 10^–5^) and were included for eQTL mapping. Of all three phenotype analyses, only the SPTB versus LTERM eQTL mapping resulted in 90 significant *cis*-eQTL transcript-SNP hits (p < 0.05) (Supplementary Fig. S4). One of these significant transcript-SNP pair, TC1100011170.hg.1-rs76196041, was significant at FDR p < 0.05 (FDR p = 0.019). Both the SNP and transcript were located in non-coding regions of chromosome 11 (see Supplementary File 5). Significant microRNA transcripts (p < 0.05) detected included microRNA 548L, microRNA 1343 and microRNA 139. A total of 219,593 trans-eQTL were highlighted for SPTB versus LTERM, of which 7 were significant at FDR p < 0.05, all of which were in either unknown regions or non-coding regions. Several trans-eQTLs were also significant at FDR p < 0.05 for the remaining phenotype comparisons: PPROM versus HTERM (n = 23), SPTB versus HTERM (n = 72), PPROM versus LTERM (n = 23) (see Supplementary File 6).

### EQTL mapping at week 20 of gestation

At week 20 of gestation only SPTB versus LTERM yielded significant *cis*-eQTLs. At p < 0.05, 91 significant *cis*-eQTLs were detected, however none of the were significant after FDR p < 0.05 (Supplementary Fig. S5). Of the 217,045 significant trans-eQTLs (p < 0.05), 43 were significant at FDR p < 0.05.

SPTB versus HTERM trans-eQTL mapping at week 20 of gestation highlighted one significant coding transcript TC1400008661.hg.1 (*TRDV3*, T cell receptor delta variable 3 on chromosome 14) in association with SNP chr1.177008414_C (rs146756455, an intron variant of *ASTN1*, Astrotactin 1, on chromosome 1) (FDR p = 0.04883). The remaining transcripts and SNPs were identified in non-coding regions. Trans-eQTLs that reached FDR p < 0.05 for the remaining phenotypes included: PPROM versus HTERM (n = 10), SPTB versus HTERM (n = 65), PPROM versus LTERM (n = 62) (see Supplementary File 6).

## Discussion

The findings of this study strongly indicate different molecular attributes of PPROM and SPTB when compared with the term phenotypes, HTERM (women with a history of preterm birth and subsequent natural term birth) and LTERM (women with successive term births).

Two genome-wide significant SNPs were detected, *ASTN1* (rs146756455, SPTB versus HTERM) (Fig. 3c) and *MAST1* (rs188343966, sPTB cases versus LTERM) (Fig. 4b). Both genes are associated with neurodevelopment disorder in preterm infants^31,32^. *ASTN1* was associated with prenatal development by Lionel et al.^31^, whereas *MAST1* was reported in preterm infants, after birth, by Arpón et al.^32^. This correlates to our findings that (1) when all sPTB cases genotypes were compared to LTERM healthy pregnancies *MAST1* was detected, (2) when the pregnancy outcomes were further stratified only SPTB genotypes were associated with *ASTN1* when compared with term pregnancies that did not have a recurrent sPTB (HTERM). This suggests that *ASTN1* is a candidate marker for the SPTB phenotype. These results also imply that perturbations in the process of neurodevelopment via various molecular pathways, which would lead to poor prognosis in preterm infants, could in fact be detected in early stages of pregnancy. Furthermore, it suggests a genetic difference between women who delivered term in all pregnancies (LTERM) and those who delivered sPTB followed by a term delivery (HTERM). This finding is novel in the obstetrics field of research. In terms of clinical management of patients, a genetic biomarker could aid risk stratification of high-risk women in a subsequent pregnancy.

The transcriptomics results suggest that local inflammation, potentially induced by TNF, occur in women who experienced PPROM, which is concordant with Capece et al.^18^ findings. The results further imply that RNA from maternal blood sampled at week 20 gestation could be utilised for predicting risk of early labour. Gene expression analysis highlighted microRNA 142 as significant in the PPROM versus HTERM outcome comparison at week 20 of gestation (FDR p = 0.02) (Table 1, Fig. 5). A PTB study of cervical cells by Sanders et al.^23^ reported increased expression of microRNA 142 (and 5 other microRNAs) in preterm cases compared to term controls, thereby associating this transcript with shorter gestational length. Sanders et al.^23^ conducted network analysis of mRNA targets of the 6 upregulated microRNA (including microRNA 142) to identify molecules involved in DNA replication and inflammatory processes. This included a key role of tumour necrosis factor (TNF), which can induce inflammation resulting in preterm delivery^33,34^. IL1RN was also detected (Table 1) and is related to early onset of labour due to infection^35–40^.

Sanders et al.^23^ also determined DNA methyltransferases in the network analysis and DNA methylation is known to supress microRNA 142, which suggests a role of epigenetics in PPROM^41^. Future work would include collecting and investigating epigenetic data from this cohort of women and comparing back to these findings for further insight on the potential mechanism of microRNAs and inflammatory response in PPROM.

The role of inflammation and immune response is also likely for women experiencing SPTB, though the different molecular signatures suggest that different mechanisms are activated compared to PPROM. SPTB versus HTERM (at week 20 of gestation) gene set enrichment analysis highlighted the significant gene set GSE37533, an “immunologic signature”. This gene set consists of PPAR-gamma, which initiates adipocyte differentiation and has been suggested to combine with Foxp3 to regulate transcriptional signatures of regulatory T-cells^42^. In addition to this immunological finding, a significantly upregulated gene set in whole blood including many inflammation-associated genes was identified (Supplementary Fig. S3). Detection of such inflammation biomarkers in the blood could lead to the development of a non-invasive screening tool for clinical practice. *Cis*-eQTL mapping highlighted one significant transcript-gene pair (FDR p < 0.05) at week 16 of gestation. No biological plausibility was identified for rs76196041 or the transcript as they were both located in non-coding regions. Rs76196041 detected in the GWAS analysis of SPTB versus LTERM (p = 8.15e−06) did not meet genome-wide significance but reached above the arbitrary suggestive threshold of p < 1 × 10^–5^ (Fig. 3). This implies that the association of rs76196041 between SPTB and LTERM could distinguish between the two phenotypes. This is particularly of importance as the LTERM group were healthy controls who never experienced sPTB. Through many trans-eQTLs were significant at both timepoint analyses, gene expression is more likely to be affected by SNPs that are closer to the gene loci therefore *cis*-eQTLs are of interest^43,44^.

One of the major strengths of the study include the unique study cohort. Women were prospectively recruited in Liverpool and applied a threshold of ≤ 34 weeks of gestation to capture clinically well-defined sPTB pregnancy outcomes. In addition to this, two control groups, LTERM healthy controls and HTERM women who delivered term after a previous sPTB. Information on recurrent pregnancies does not exist in other biobanks. Another key strength was that both genomic and transcriptomic data were obtained from the same women (at two timepoints), and this was investigated using unbiased genome and transcriptome profiling technologies to detect molecular signatures. This provided an insight into the interactions between the omic layers and determine potential mechanistic pathways, which is a benefit of multi-omic studies.

Due to the clear definition of each sPTB group, the numbers of cases were low in this study. Therefore, differences in gene expression between PPROM and SPTB could not be detected. However, when these cases were compared with term births, molecular signatures were detected, indicating that there are genetic differences between SPTB and PPROM when compared to term birth outcomes. This limitation was also present for the GWAS analyses; however, the detection of genome-wide significant SNPs warrants further investigation for instance with PCR methods. Furthermore, LTERM samples could not be processed on the Clariom D array, due to limited funds. An advantage of eQTL mapping was that it allowed for SNPs from LTERM GWAS analyses to be correlated to expression data of HTERM or sPTB births.

## Conclusion

This study identified multiple inflammation biomarkers of sPTB in a unique, well-defined cohort of women who delivered ≤ 34 weeks of gestation. This study has also demonstrated the potential for multi-omics biomarkers as a diagnostic tool to detect if a woman is at risk of delivering preterm and thereby enabling early clinical intervention. Future work involves validation of the genes and transcripts identified in our cohort samples. Integration of this data with other omics, such as metabolomics would direct this analysis towards identification of potential pathways involved with the initiation of PTB.

## Methods

### Study participants

Women were recruited at 16 and 20 weeks of gestation in a subsequent singleton pregnancy at the Liverpool Women’s Hospital Preterm Birth Prevention Clinic, between April 2012 and December 2017. An additional singleton ‘low risk for PTB’ pregnancy population with a history of only term births (≥ 39 weeks of gestation) were also recruited at 16 and 20 weeks. Participants were included in the study if they were > 18 years old, willing to undergo transvaginal ultrasound scan and were able to provide written consent. For women ‘high-risk of PTB’, a further inclusion criterion of previous PPROM or SPTB (> 16 and ≤ 34 weeks of gestation). The exclusion criteria are reported in Fig. 1.

Study sample size and power calculations were conducted based on data obtained from the recruitment of a pilot cohort of women. Further details are provided in Supplementary Methods and Supplementary Fig. S6 (see Supplementary File 1). All pregnancies were followed up and clinical data were collected. Informed consent was obtained from participants. Research ethics approval for this nested case–control study was obtained from the North West Liverpool Central Research Ethics Committee (REC reference: 11/NW/0720). This study was conducted in accordance with institutional and national ethical standards and complied with the 1964 Helsinki Declaration and its later amendments or comparable ethical standards.

Participants were categorised into mutually exclusive phenotypes based on their current pregnancy outcome. For women considered high risk of PTB (history of PTB < 34 weeks): HTERM (birth ≥ 37^+0^ weeks) or SPTB (spontaneous preterm birth) or PPROM (preterm premature rupture of the membranes with > 12 h prior to labour onset) (PTB ≤ 34^+0^ weeks gestation). Low-risk women with a recurrent term pregnancy (≥ 39^+0^ weeks), were our control group, labelled LTERM and represented normality.

Preterm cases, SPTB and PPROM, were combined as a “PTB cases < 34 week” group and compared against both high risk (HTERM) and low risk controls (LTERM) to determine if statistical power in our GWAS was increased.

### Sample preparation, quality checks and microarrays

Whole blood samples were collected in BD vacutainer® K_2_EDTA tubes. DNA was extracted using the Chemagenic Magnetic Separation Module I (Auto Q Biosciences Ltd, UK) from maternal whole blood (N = 369). Oxford Genomics Centre at the Wellcome Centre for Human Genetics processed the DNA samples on the Applied Biosystems™ UK Biobank Axiom™ array (Thermo Fisher Scientific) for genome-wide screening.

Further whole blood samples were collected in PAXgene Blood RNA Tubes (PreAnalytiX, QIAGEN) and stored at − 80 °C. Total RNA (N = 115) was extracted using spin column PreAnalytix kit (PreAnalytiX, QIAGEN). Sample quality was measured using the RNA 6000 Nano and Pico Kit and Agilent 2100 BioAnalyzer (Agilent Technologies). Samples with RNA integrity number (RIN) of > 7 were hybridised to the GeneChip™ Clariom™ D Human assay (Thermo Fisher Scientific).

Data generated from the array platforms have been deposited at the European Genome-phenome Archive (EGA)^45^, which is hosted by the EBI and the CRG, under the study accession number EGAS00001005076.

### GWAS data quality control

Genotyping with the Axiom™ array was performed and Axiom™ Analysis Suite 2.0 was applied to identify samples that passed 97% genotype call rate by Oxford Centre for Genomics (Wellcome Centre for Human Genetics, University of Oxford). GWAS standard quality control procedures reported by Anderson et al.^46^ and Marees et al.^47^ were followed using PLINK v1.9 software^48^.

Samples were excluded due to the following reasons: overall heterozygosity rate was ± 3 standard deviations from the cohort mean a high proportion of missing SNPs; heterozygosity on chromsome X resulting in gender discrepancy or indication of close relatedness (or ‘identical by descent’, IBD) with other samples. Samples were included if genetically assigned to European ancestry (CEU) population based on the HapMap data^49,50^ (Supplementary Fig. S7).

SNPs were excluded if they had a low genotype call rate (< 95%); minor allele frequency (MAF) of < 1% and if SNPs deviated from Hardy–Weinberg Equilibrium (HWE) at p ≤ 1 × 10^–6^^47^. Prior to imputation, HRC Perl script v4.2.7 by Will Rayner was executed to remove trialleic, biallelic and palindromic SNPs.

Phasing and imputation of 618,283 SNPs was completed using the Michigan Imputation Server, applying Eagle v2.3 to phase chromosome 1 to 22 and minimac3 algorithm with the HRC r1.1 2016 reference panel for imputation^51,52^. Variants with R^2^ < 0.3^53^ and MAF = 0 (or < 1%) were excluded post-imputation to retain higher quality imputated SNPs.

### GWAS analysis

Frequentist association analysis between the four individual phenotypes was completed using SNPTEST v2.5^54–56^. Multi-dimensional scaling components 1 to 6 were included as covariates to control for genetic variance observed in the cohort. Manhattan plots were generated using R package ‘qqman’^28^. Biomart Ensembl GRCh37 (release 97, EMBL-EBI), was applied for SNP annotation (SNPs reaching p < 1 × 10^–5^ and genome-wide significance of p < 5 × 10^–8^)^57^. Regional plots were generated using LocusZoom (University of Michigan) web tool to explore SNPs in linkage disequilibrium^29^.

### Differential gene expression analysis

Array data was pre-processed by performing Robust Multichip/multi-array Analysis (RMA) using the R Bioconductor package, ‘oligo’^58^. Annotation was completed with R package ‘affycoretools’^59^ and the Clariom D Human array database ‘pd.clariom.d.human’^60^. Differential gene expression analysis was conducted on SPTB, PPROM and HTERM outcome comparisons (at both week 16 and 20 of gestation) using R package ‘limma’ with ANOVA and empirical Bayes^30^.

### Pathway analysis

Gene symbols of significant DEGs (FDR p < 0.05) from PPROM cases versus HTERM controls gene expression analysis were uploaded onto Reactome^61,62^ for pathway analysis. Missing gene symbols were excluded.

### Gene set enrichment

FUMA GWAS online software (Functional Mapping and Annotation of Genome-Wide Association Studies) was applied for gene set enrichment analysis (GSEA, Broad Institute, US) of genes identified from expression analysis across all phenotypes (using p < 0.05 threshold)^63^. Genotype-Tissue Expression (GTEx) database was used to identify whether these transcripts could impact tissue-specific gene expression.

### Matrix eQTL analysis

R package ‘MatrixEQTL’^43^ was applied for eQTL mapping of the gene expression data and SNPs with p < 1 × 10^–5^ from GWAS analyses comparing PPROM versus HTERM, PPROM versus LTERM, SPTB versus HTERM, SPTB versus LTERM. Linear regression model was applied, with thresholds of p < 0.02 for *cis*-eQTLs (local) and p < 0.01 for trans-eQTLs (distant). The test statistics for every transcript-SNP pair exceeding the threshold were returned with the corresponding p-values.

### Ethics approval

Research ethics approval was Granted by the North West Research Ethics Committee (REC reference: 11/NW/0720).

### Consent to participate

All participants provided informed consent for this study.

## Supplementary Information

Supplementary Information 1.

Supplementary Information 2.

Supplementary Information 3.

Supplementary Information 4.

Supplementary Information 5.

Supplementary Information 6.

## Data Availability

The genomic and transcriptomic datasets generated and/or analysed during the current study are available in the European Genome-Phenome Archive (EGA) EBI repository (https://ega-archive.org/studies/EGAS00001005076).
