# Publisher Correction: Genome and transcriptome profiling of spontaneous preterm birth phenotypes

**DOI:** 10.1038/s41598-022-06338-w

**Published:** 2022-02-01

**Authors:** Juhi K. Gupta, Angharad Care, Laura Goodfellow, Zarko Alfirevic, Bertram Müller‑Myhsok, Ana Alfirevic

**Affiliations:** 1 Wolfson Centre for Personalised Medicine, Department of Pharmacology and Therapeutics, Institute of Systems, Molecular and Integrative Biology, University of Liverpool, Block A: Waterhouse Buildings, 1‑5 Brownlow Street, Liverpool, L69 3GL UK; 2 Harris‑Wellbeing Research Centre, University Department, Liverpool Women’s Hospital, Liverpool, L8 7SS UK; 3 Max Planck Institute of Psychiatry, 80804 Munich, Germany

Correction to: *Scientific Reports * 10.1038/s41598-022-04881-0, published online 19 January 2022

In the original version of the Article, Figure 1 was a duplication of Figure 2.

The original Article has been corrected.

