# Supplementary material for: Genome and transcriptome profiling of spontaneous preterm birth phenotypes
Source: Sci Rep. 2022 Jan 19;12:1003. doi: 10.1038/s41598-022-04881-0 (PMC8770724; doi:10.1038/s41598-022-04881-0)
Supplement: Supplementary file 1 — Supplementary Information 1. [file 41598_2022_4881_MOESM1_ESM.pdf]

Supplementary Information: File 1

Genome and transcriptome profiling of spontaneous preterm birth phenotypes

Juhi K. Gupta[1,2]*, Angharad Care[2], Laura Goodfellow[2], Zarko Alfirevic[2], Bertram Müller-Myhsok[1, 3], Ana Alfirevic[1,2]

[1] Wolfson Centre for Personalised Medicine, Department of Pharmacology and Therapeutics, Institute of Systems, Molecular and Integrative Biology, University of Liverpool, Liverpool, L69 3GL

[2] Harris-Wellbeing Research Centre, University Department, Liverpool Women's Hospital, Liverpool, L8 7SS

[3]Max Planck Institute of Psychiatry, 80804, Munich, Germany

* J.Gupta@liverpool.ac.uk

Table of contents

Supplementary Figures

Figure S1. Manhattan plot of spontaneous preterm births and low-risk term births GWAS analysis.

Figure S2. Manhattan plot of preterm birth cases and high-risk term births GWAS analysis.

Figure S3. Schematic of differentially expressed gene sets in week 20 of gestation (HTERM-SPTB).

Figure S4. Q-Q plot of eQTLs identified at week 16 of gestation (SPTB-LTERM).

Figure S5. Q-Q plot of eQTLs identified at week 20 of gestation (SPTB-LTERM).

Supplementary Methods

Figure S6. Study power calculation and ROC curves.

Figure S7. Genomics population stratification principal component plot.

Supplementary Figures

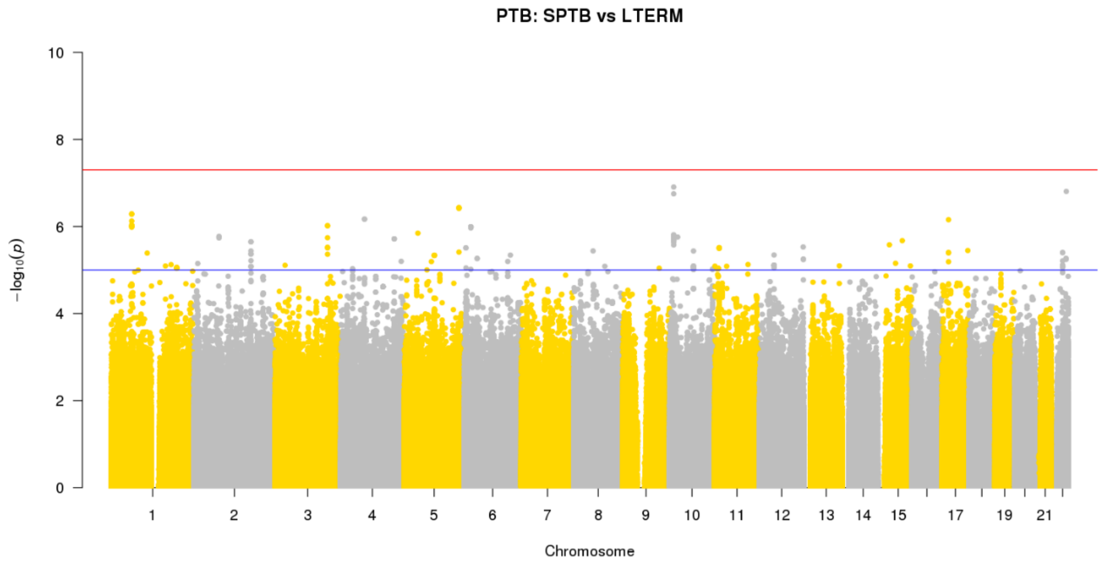

Supplementary Fig. S1. Manhattan plot of spontaneous preterm births (SPTB) and low-risk term (LTERM) GWAS analysis. SPTB ≤ 34 weeks gestation (n=23) and LTERM (n=160) were included in the Frequentist association analysis. Multi-dimensional scaling (MDS) components 1 to 6 of the cohort were included as covariates. The vertical red line is genome-wide significance threshold, $5x10^{-8}$; the blue line represents a suggestive threshold of $1x10^{-5}$. No genome-significant SNPs were observed in this comparison. This plot was generated using R package 'qqman' [1].

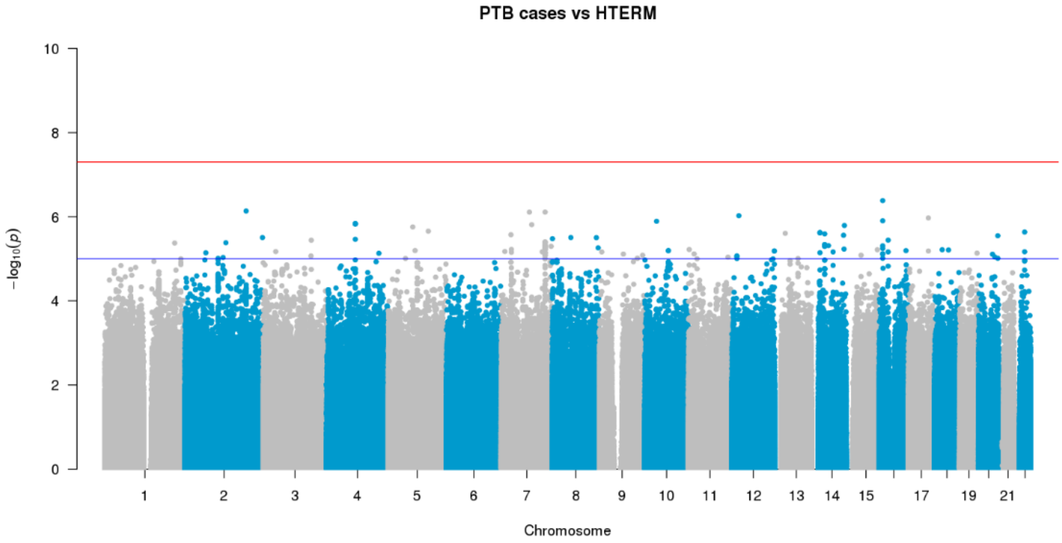

Supplementary Fig. S2. Manhattan plot of all preterm birth cases and high-risk term (HTERM) GWAS analysis. PTB cases (≤ 34 weeks gestation, n=48) and HTERM births (n=102) were included in the Frequentist association analysis. Multi-dimensional scaling (MDS) components 1 to 6 of the cohort were included as covariates. The vertical red line is genome-wide significance threshold, $5x10^{-8}$; the blue line represents a suggestive threshold of $1x10^{-5}$. No genome-significant SNPs were observed in this comparison. This plot was generated using R package ‘qqman’ [1].

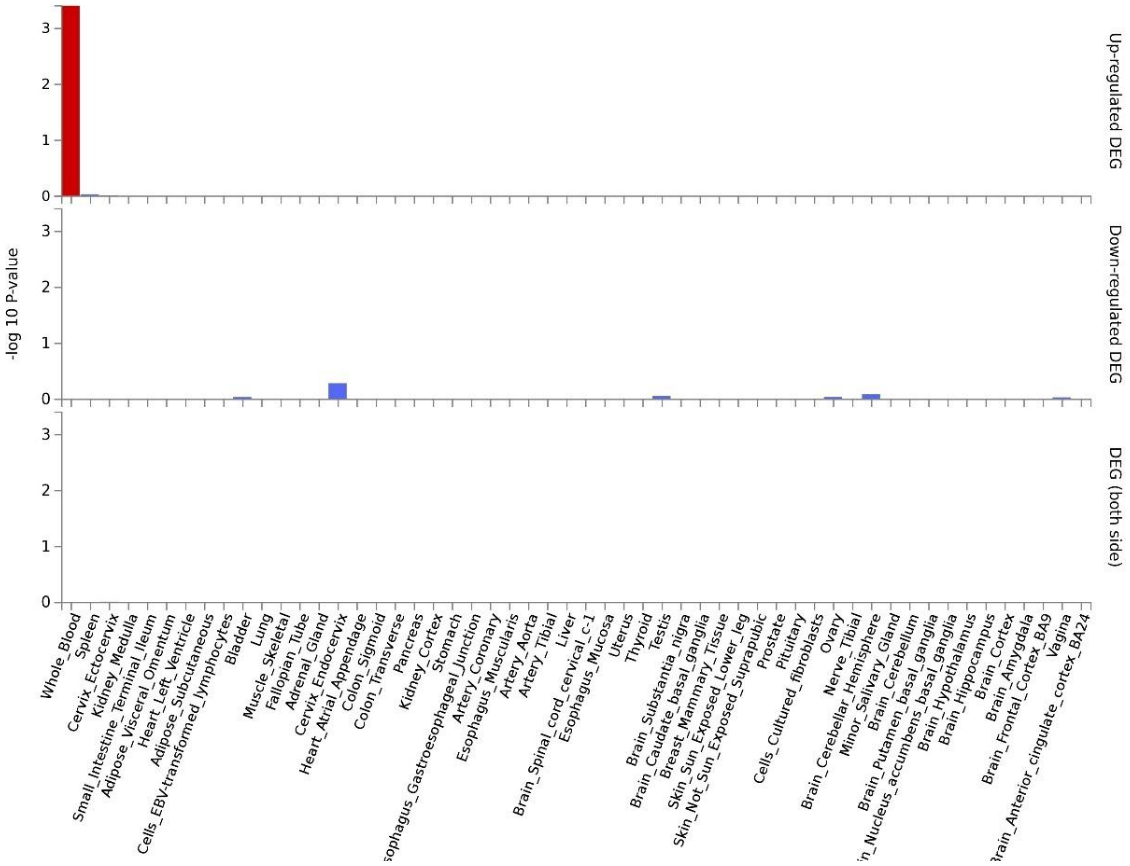

Supplementary Fig. S3. Schematic of differentially expressed gene sets in week 20 of gestation SPTB-HTERM. DEGs were defined by two-sided t-tests applied per label versus all remaining tissues from the GTEx v8 database in FUMA GWAS [2]. DEG sets in whole blood was significantly enriched (Bonferroni $p<0.05$) and upregulated. Gene sets were downregulated in tissues including cervix/endocervix, ovary and vagina.

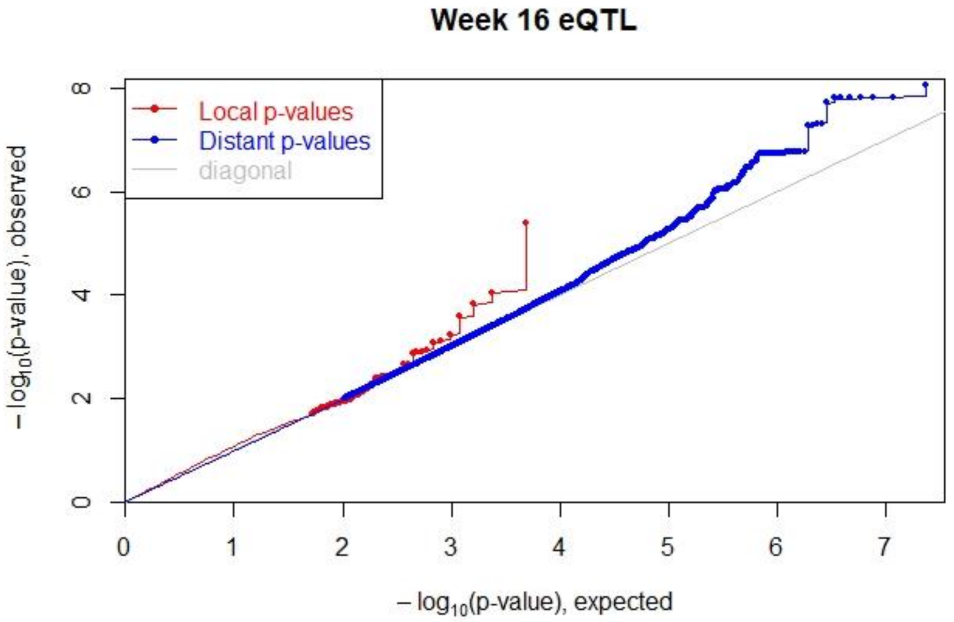

Supplementary Fig. S4. Q-Q plot of eQTLs identified at week 16 of gestation from SPTB-LTERM analysis. Both cis-eQTLs (n=90) and trans-eQTLs (n=219593) were highlighted from GWAS SNPs. Analysis was implemented using R package 'Matrix eQTL' [3].

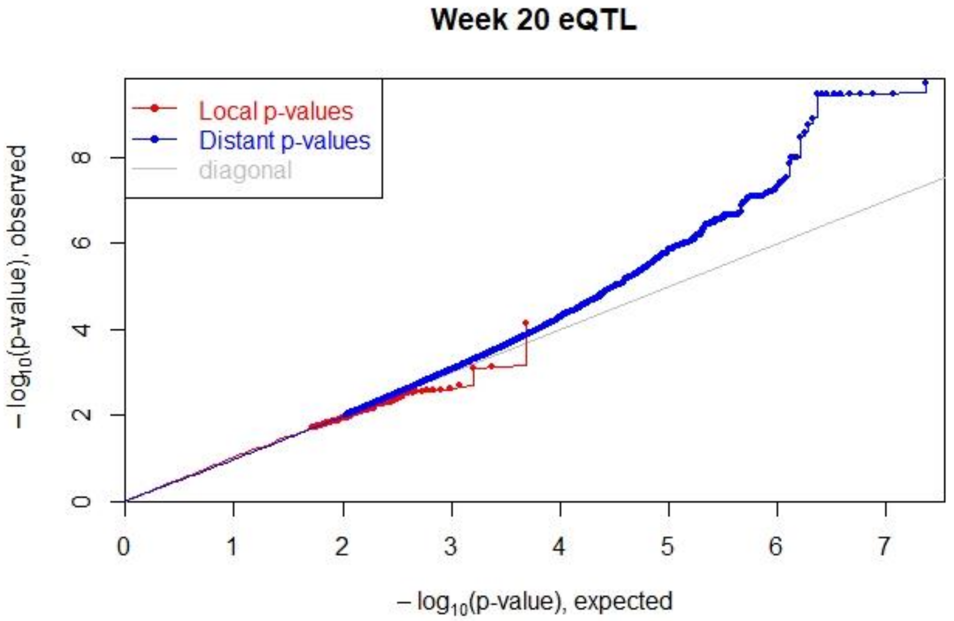

Supplementary Fig. S5. Q-Q plot of eQTLs identified at week 20 of gestation from SPTB-LTERM analysis. Both cis-eQTLs (n=91) and trans-eQTLs (n=217045) were highlighted from GWAS SNPs. Analysis was implemented using R package 'Matrix eQTL' [3].

Supplementary Methods

Sample size was calculated based on the occurrence of PTB recorded by clinical audits at the Liverpool Women's Hospital, which reported the rates as between 17-20%. Approximately 140 women could be recruited in 3 years as demonstrated by pilot recruitment of pregnant women in Liverpool. Based on the assumption that 80% of term births (controls) are recruited, an AUC of 0.9 can be achieved with 50 samples.

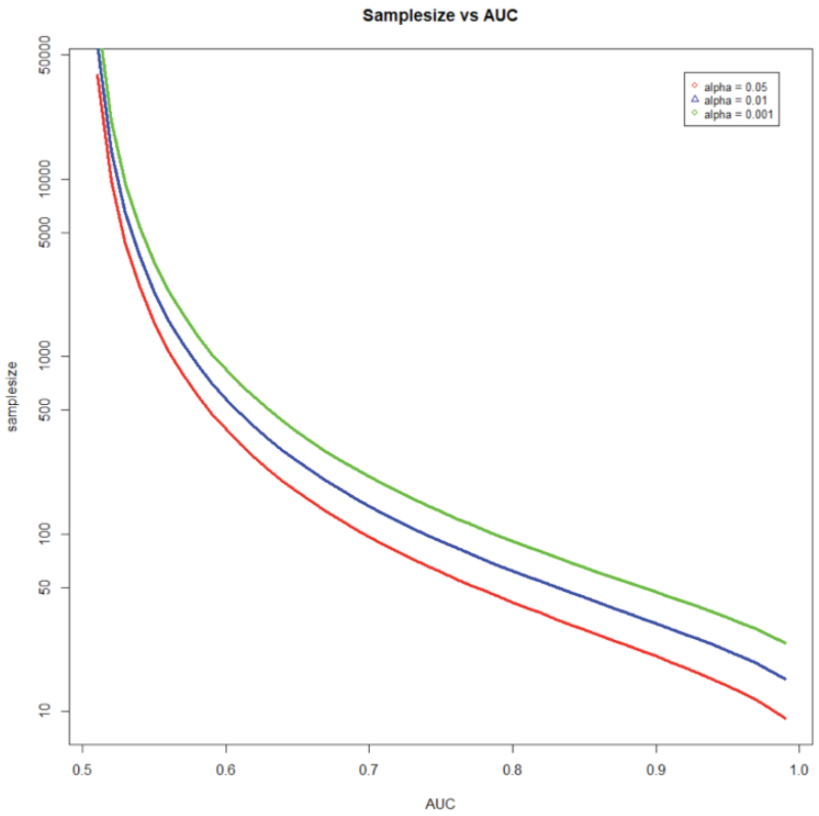

Supplementary Fig. S6. Liverpool preterm birth cohort study power calculation for multi-omic investigations. Seven machine learning prediction models, based on 80% term control samples, were performed to achieve predictive AUC of 0.9 for 50 samples at alpha = 0.001. ROC curves were generated using the R package 'pROC' [4].

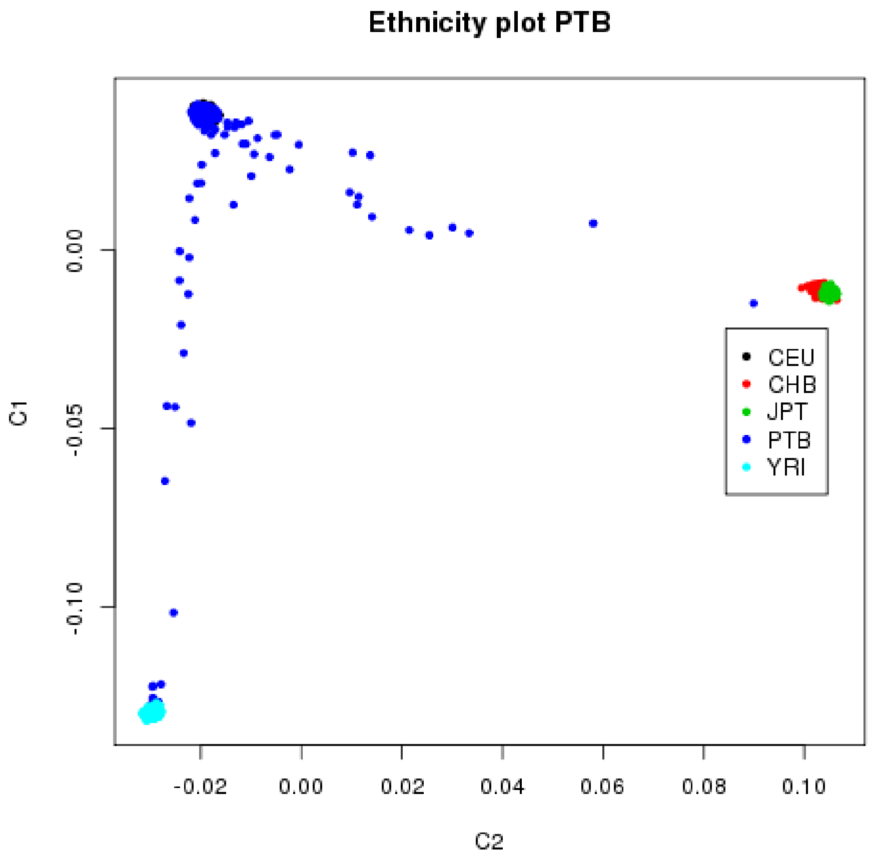

Supplementary Fig. S7. Preterm birth cohort population stratification. Preterm birth cohort GWAS merged with International HapMap Consortium continental ethnic groups, at first (C1) and second (C2) principal components of Multidimensional Scaling (MDS). The majority of the PTB participants (dark blue points) overlap with the Caucasian cluster (CEU) in the top left corner (black points). Key: PTB = preterm birth, Liverpool cohort; CEU = Utah Residents (CEPH) with Northern and Western European Ancestry; CHB = Han Chinese in Beijing, China; JPT = Japanese in Tokyo, Japan; YRI = Yoruba in Ibadan, Nigeria.

References

[1] Turner SD. qqman: an R package for visualizing GWAS results using Q-Q and manhattan plots. *Journal of Open Source Software* **3**, 731 (2018).

[2] Watanabe K, Taskesen E, van Bochoven A, Posthuma D. Functional mapping and annotation of genetic associations with FUMA. *Nat Commun.* **8**, 1826 (2017).

[3] Shabalin AA. Matrix eQTL: ultra fast eQTL analysis via large matrix operations. *Bioinformatics* **28**, 1353-8 (2012).

[4] Robin X, Turck N, Hainard A, Tiberti N, Lisacek F, Sanchez JC, et al. pROC: an open-source package for R and S+ to analyze and compare ROC curves. *BMC Bioinformatics* **12**, 77 (2011).
